# The impact of insulin resistance on the association between metabolic syndrome and lung function: the Kangbuk Samsung Health Study

**DOI:** 10.1186/s13098-023-01042-9

**Published:** 2023-04-01

**Authors:** Jonghoo Lee, Hye Kyeong Park, Min-Jung Kwon, Soo-Youn Ham, Hyun-Il Gil, Si-Young Lim, Jae-Uk Song

**Affiliations:** 1 Department of Internal Medicine, Jeju National University Hospital, Jeju National University School of Medicine, Jeju, Republic of Korea; 2 Division of Pulmonary and Critical Care Medicine, Department of Internal Medicine, Ilsan Paik Hospital, Inje University College of Medicine, Ilsan, Republic of Korea; 3 Department of Laboratory Medicine, Kangbuk Samsung Hospital, Sungkyunkwan University School of Medicine, Seoul, Republic of Korea; 4 Department of Radiology, Kangbuk Samsung Hospital, Sungkyunkwan University School of Medicine, Seoul, Republic of Korea; 5 Division of Pulmonary and Critical Care Medicine, Department of Internal Medicine, Kangbuk Samsung Hospital, Sungkyunkwan University School of Medicine, 29 Saemunan-ro, Jongno-gu, 03181 Seoul, Republic of Korea

**Keywords:** Lung function, Metabolic syndrome, Insulin resistance, Spirometry

## Abstract

**Background/Objective:**

Metabolic syndrome (MS) is related to lung dysfunction. However, its impact according to insulin resistance (IR) remains unknown. Therefore, we evaluated whether the relation of MS with lung dysfunction differs by IR.

**Subject/Methods:**

This cross-sectional study included 114,143 Korean adults (mean age, 39.6 years) with health examinations who were divided into three groups: metabolically healthy (MH), MS without IR, and MS with IR. MS was defined as presence of any MS component, including IR estimated by HOMA-IR ≥ 2.5. Adjusted odds ratios (aORs) and 95% confidence intervals (CIs) for lung dysfunction were obtained in MS, MS without IR, and MS with IR groups compared with the MH (reference) group.

**Results:**

The prevalence of MS was 50.7%. The percent predicted forced expiratory volume in 1 s (FEV1%) and forced vital capacity (FVC%) showed statistically significant differences between MS with IR and MH and between MS with IR and MS without IR (all *P* < 0.001). However, those measures did not vary between MH and MS without IR (P = 1.000 and P = 0.711, respectively). Compared to MH, MS was not at risk for FEV1% < 80% (1.103 (0.993–1.224), P = 0.067) or FVC% < 80% (1.011 (0.901–1.136), P = 0.849). However, MS with IR was clearly associated with FEV1% < 80% (1.374 (1.205–1.566) and FVC% < 80% (1.428 (1.237–1.647) (all p < 0.001), though there was no evident association for MS without IR (FEV1%: 1.078 (0.975–1.192, P = 0.142) and FVC%: 1.000 (0.896–1.116, p = 0.998)).

**Conclusion:**

The association of MS with lung function can be affected by IR. However, longitudinal follow-up studies are required to validate our findings.

¶ Jonghoo Lee, Hye Kyeong ParkThese authors contributed equally to this work.

## Introduction

Lung dysfunction is associated with frailty, breathlessness, and multimorbidity, including death from all causes and cardiovascular diseases [1–3]. As lung dysfunction can occur prior to overt disease manifestation [4], early identification of modifiable risk factors for lung dysfunction is meaningful to reduce various diseases and their complications. Lung function is adversely affected by metabolic syndrome (MS) [5, 6], which comprises modifiable cardio-metabolic abnormalities, including abdominal obesity, hyperglycemia, hypertension, dyslipidemia, and/or insulin resistance (IR) [7, 8]. Furthermore, the extent to which MS affects lung function depends on the numbers [9–15] and components [9–26] that are criteria for MS. Therefore, the role of MS in lung function is an area of active scrutiny, considering the worldwide high prevalence and modifiable precondition of MS-associated major critical health problems [5].

However, there have been variable results on the association between MS and lung function due to multiple concurrent definitions of MS, as suggested by a previous study [27]. IR critically mediates the link between lung dysfunction and MS [5, 22, 24, 28]. However, the label of MS per se does not provide a sensitive approach to identifying IR [29]. Thus, omission of IR as a criterion for MS could increase uncertainty as to MS-related lung effects. Nevertheless, among MS components, IR has not been fully considered in any previous studies. Therefore, we investigated spirometric values in subjects of different metabolic health and IR status to investigate the impact of IR on MS-related lung effects in a large asymptomatic population.

## Materials and methods

### Study design and population

This cross-sectional study was a part of the Kangbuk Samsung Health Study, which involved Koreans who underwent a comprehensive health examination at the Total Healthcare Center of Kangbuk Samsung Hospital in Seoul and Suwon, Republic of Korea, since January 1, 2002. Most of the examinees were employees and family members of various companies or local governmental organizations. In Republic of Korea, annual or biennial employee health screenings are required by the Industrial Safety and Health Law and are free of charge.

**Fig. 1:**
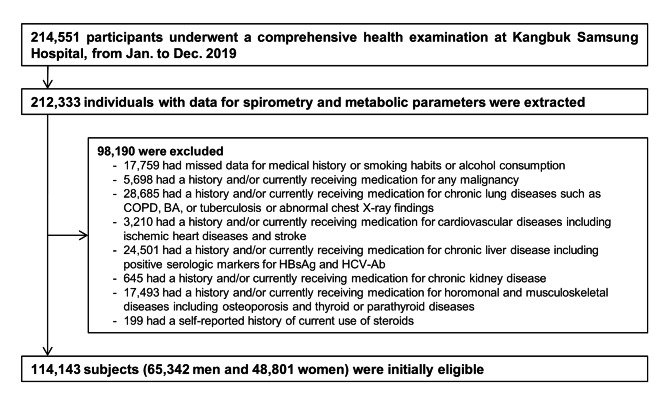
Flow diagram of study participants BA = bronchial asthma; COPD = chronic obstructive pulmonary disease; HBsAg = hepatitis B virus surface antigen; HCV-Ab = hepatitis C virus antibody

Figure 1 shows the flow chart for inclusion and exclusion of subjects in analyses. This study began with data from 214,551 individuals with health examinations in 2019. From these subjects, inclusion criteria were participants aged 18 years or older with recorded spirometry and metabolic data used to ascertain metabolic health (N = 212,333). Among this cohort, we excluded subjects with missing data for medical history and smoking habits or alcohol consumption (n = 17,759). We additionally excluded participants with a self-reported history and/or those patients currently receiving medication for malignancy (n = 5,698), chronic lung disease or abnormal chest radiograph findings ( n = 28,685), cardiovascular or cerebrovascular disease (n = 3,210), chronic liver disease including positive hepatitis B surface antigen and anti-hepatitis C virus antibody (n = 24,501), chronic renal disease (n = 645), hormonal and musculoskeletal diseases including osteoporosis and thyroid or parathyroid diseases (n = 17,493), and current steroid use (n = 199). However, detailed comorbidities were unavailable (not specified) because the medical history questionnaire only required yes/no responses. As some individuals had more than one exclusion criterion,

114,143 participants were ultimately included in the analysis (Fig. 1).

The study was approved by the Institutional Review Board of Kangbuk Samsung Hospital (KBSMC 2022-03-055), which waived the requirement for informed consent due to the use of de-identified data for analysis purposes. The study protocol conformed to ethical guidelines of the 1975 Declaration of Helsinki and followed the Strengthening the Reporting of Observational Studies in Epidemiology (STROBE) reporting guideline.

### Data collection, anthropometric measurements, and laboratory tests

The comprehensive health-screening program assessed demographic, anthropometric, and laboratory data. Standardized self-administered questionnaires were used to ascertain information on demographic characteristics, medical history, medication use, smoking and drinking habits, exercise frequency, and education level. However, detailed comorbidities were unavailable because the medical history questionnaire only required yes/no responses.

Smoking status was classified as nonsmoker, ex-smoker, or current smoker. Alcohol consumption was categorized as none, non-heavy (≤ 20 g/day), or heavy (> 20 g/day). Weekly frequency of moderate physical activity (defined as more than 30 min of activity per day inducing slight breathlessness) was also assessed, and regular exercise was defined as ≥ 3 times/week [30]. Education level was categorized as less than college graduate or college graduate or more [30]. Diabetes mellitus and hypertension were defined as ever diagnosis with these diseases or presently taking related medications.

Physical characteristics and serum biochemical parameters were measured by trained nurses as previously reported [22, 30]. Height and weight were measured with individuals wearing a lightweight hospital gown and bare feet, using automated instruments (InBody 3.0 and Inbody 720, Biospace Co., Seoul, Republic of Korea) that were validated for reproducibility and accuracy of body composition measurements [31] and were calibrated every morning before testing. BMI was calculated by dividing weight (kg) by the square of height (m^2^). Obesity was defined as BMI ≥ 25 kg/m^2^ [32]. Blood pressure (BP) was measured with a standard sphygmomanometer after at least 5 min of seated rest. Measurements were performed twice at 5-min intervals and were averaged for analysis.

After at least a 10-h fast, a blood sample was drawn for measurement of liver enzymes, creatinine, lipid profiles, glucose, glycated hemoglobin (HbA1c), insulin, and high-sensitivity C-reactive protein (hsCRP). Serum total cholesterol and triglycerides were determined with an enzymatic colorimetric assay. Low-density lipoprotein cholesterol (LDL-C) and high-density lipoprotein cholesterol (HDL-C) were determined through a homogeneous enzymatic colorimetric test. Serum glucose was measured using the hexokinase method on a Cobas Integra 800 apparatus (Roche Diagnostics). HbA1c was measured using an immunoturbidimetric assay with a Cobra Integra 800 automatic analyzer (Roche Diagnostics).

Serum insulin levels were measured using an electrochemiluminescence immunoassay on a Modular Analytics E170 apparatus (Roche Diagnostics, Tokyo, Japan). Serum hs-CRP levels were measured using a nephelometric assay (BNII nephelometer, Dade Behring, Deerfield, IL, USA). The inter- and intra-assay coefficients of variation for quality control specimens were < 5% for the blood variables. IR was assessed using the homeostasis model assessment of insulin resistance (HOMA-IR) equation: fasting blood insulin (µU/ml) × fasting blood glucose (mmol/l)/22.5 [33]. The Laboratory Medicine Department at Kangbuk Samsung Hospital is accredited and participates annually in inspections and surveys by the Korean Association of Quality Assurance for Clinical Laboratories.

### Definition of metabolic health and insulin resistance

We selected metabolically healthy subjects using metabolic syndrome components of harmonized criteria [2]. Because risk for health implications of metabolic syndrome such as lung dysfunction [8–13, 17] and all-cause or cardiovascular mortality [34] increased incrementally, beginning at 1 risk factor, a very strict definition of metabolic health could be necessary to define the ideally healthy group as a reference to provide a very clear test to determine the effects of MS on lung function impairment that was missed in the previous studies. Therefore, metabolically healthy (MH) was defined as having none of the following metabolic abnormalities [7, 8]: (1) fasting glucose level (FBS) ≥ 100 mg/dL or on medications for diabetes, (2) BP ≥ 130/85 mmHg or on anti-hypertensive drugs, (3) triglyceride (TG) level ≥ 150 mg/dL or on lipid-lowering drugs, (4) high-density lipoprotein cholesterol (HDL-C) < 40 mg/dL in men or < 50 mg/dL in women, and (5) IR defined as HOMA-IR score ≥ 2.5 [35]. In contrast, metabolic syndrome (MS) was defined as having one or more of these metabolic abnormalities. According to these criteria, participants were divided into three groups: MH, MS without IR, and MS with IR.

### Lung function measurement

Spirometry was performed according to the American Thoracic Society/European Respiratory Society guidelines [36], using the Vmax22 system (Sensor-Medics, Yorba Linda, CA, USA). Forced expiratory volume in 1s (FEV_1_) and forced vital capacity (FVC) were obtained under a pre-bronchodilatory setting. The highest FEV1 and FVC values from three or more tests with acceptable curves were used for analyses. The predicted values for FEV1 and FVC were calculated using equations for a representative Korean population sample [37]. The predicted FEV1% (FEV1%) and FVC% (FVC%) were calculated by dividing the measured value (L) by the predicted value (L) and converting the quotient into a percentage. The ratio of FEV1 to FVC (FEV1/FVC) was calculated using the actual measurements. The following criteria were used to determine impaired lung function: FEV1% <80%, FVC% <80%, and FEV1/FVC < 0.7 (obstructive lung function, OLF) [36].

### Statistical analyses

Data are expressed as mean ± standard deviation (SD) or median and interquartile range (IQR) for continuous variables and as number (%) for categorical variables. The normality of continuous variables was assessed with the Kolmogorov-Smirnov test. The baseline continuous variables were stratified by metabolic health and IR status and were compared using one-way analysis of variance (ANOVA) or Kruskal-Wallis test. Chi-square test or Fisher’s exact test was used for categorical variables.

Analysis of covariance (ANCOVA) was performed to test differences in mean values of lung function parameters between the three groups divided by metabolic health and IR status after adjusting for age, sex, and continuous variables with *P <* 0.05 in univariate analyses. Post-hoc analysis was performed using the Bonferroni correction to compare the mean spirometric values between study groups.

All covariates were transformed into categorical variables to analyze the significance of differences between the three groups: high or low and with or without. Differences among the three groups were tested using Chi square or Fisher’s exact test. Multivariate analysis using binary logistic regression was conducted to examine the impact of IR on MS-related lung effects. Adjusted odds ratios (aORs) with 95% confidence intervals (CIs) were estimated to determine the risk for lung function impairment in MS, MS without IR, and MS with IR compared with MH (reference). We used three models to progressively adjust for potential confounders: model 1 was adjusted for age, sex, center, smoking status, alcohol intake, regular exercise, and education level; model 2 was adjusted as in model 1 plus for metabolic components of BMI, total cholesterol, low-density lipoprotein cholesterol (LDL-C), and HbA1c; model 3 was adjusted as in model 2 plus for hsCRP and variables with *P <* 0.05 in univariate analyses. As FVC (L) and FEV1 (L) were strongly correlated (r = 0.942, *P <* 0.001), they were assessed separately to avoid confounding effects. All tests were two-sided, and *P* values *<* 0.05 were considered statistically significant. Data were analyzed using IBM SPSS Statistics 24.0 (IBM Corp., Armonk, NY, USA).

## Results

### Baseline characteristics of participants

The baseline characteristics of the 114,143 eligible subjects (57.2% male, 39.6 ± 7.8 years), stratified into three groups (MH, MS without IR, and MS with IR) are shown in Table 1. Classification of subjects according to metabolic health and IR status showed that 49.3% were MH, 35.3% were MS without IR, and 15.4% were MS with IR. The prevalence of MS was 50.7%, the mean BMI was 23.6 ± 3.3 kg/m^2^, and 57.4% of subjects were non-smokers. Of the study population, 2.0% had diabetes and 7.2% were hypertensive. MS groups were older and more likely to be men, to have smoked, and to drink alcohol. Specifically, subjects in the MS with IR group had the worst levels of TG, HDL-C, fasting glucose, HbA1c, insulin, hsCRP, and blood pressure (BP) among the groups (*P* < 0.001). Also, the values of FEV1, FEV1%, FVC, and FVC% were significantly lower in the MS with IR group than in the other two groups (*P* < 0.001). However, the MS without IR group included the highest proportion of subjects who exercised regularly.

**Table 1:** Baseline characteristics of study participants classified by metabolic health and IR status

	All Subjects (n = 114,143)	MH (n = 56,311, 49.3%)	MS without IR (n = 40,255, 35.3%)	MS with IR (n = 17,577, 15.4%)	*p* value^*^
Age (years)	39.6 ± 7.8	37.8 ± 7.2	42.0 ± 8.0	39.6 ± 7.9	< 0.001
Sex (male)	65,342 (57.2)	23,430 (41.6)	29,751 (73.9)	12,161 (69.2)	< 0.001
Center (Seoul)	56,201 (49.2)	29,434 (52.3)	20,179 (50.1)	6,588 (37.5)	< 0.001
Height (cm)	168.2 ± 8.4	166.5 ± 8.3	169.8 ± 8.1^a^	170.0 ± 8.3 ^a^	< 0.001
Weight (kg)	67.1 ± 13.1	61.8 ± 11.2	71.0 ± 11.4	77.7 ± 13.9	< 0.001
BMI (kg/m^2^)	23.6 ± 3.3	22.1 ± 2.7	24.2 ± 2.8	26.8 ± 3.7	< 0.001
Smoking status					< 0.001
Non smoker	6,483 (57.4)	39,504 (70.2)	17,520 (43.5)	8,459 (48.1)	
Ex-smoker	29,669 (26.0)	10,641 (18.9)	13,639 (33.9)	5,3889 (30.7)	
Current smoker	18,991 (16.6)	6,166 (10.9)	9,096 (22.6)	3,729 (21.2)	
Smoking (pack-years)	3.8 ± 7.3	2.1 ± 5.1	5.7 ± 8.7	5.2 ± 8.5	< 0.001
Alcohol intake	104,986 (92.0)	51,572 (91.6)	37,156 (92.3)	16,258 (92.5)	< 0.001
No alcohol	9,157 (8.0)	4,739 (8.4)	3,099 (7.7)	1,319 (7.5)	
Amount of alcohol consumption (g/day)	13.9 ± 21.5	10.6 ± 17.4	17.3 ± 24.5	16.2 ± 24.0	< 0.001
Moderate physical activity frequency (times/week) (n = 113,742)	0.87 ± 1.42	0.87 ± 1.43	0.94 ± 1.46	0.71 ± 1.26	< 0.001
Regular exercise (≥ 3 times/week) (n = 113,742)	15,346 (13.5)	7,743 (13.8)	5,863 (14.6)	1,740 (9.9)	< 0.001
High education (≥ college graduate) (n = 111,747)	94,181 (84.3)	47,630 (86.2)	32,687 (83.1)	13,864 (80.7)	< 0.001
Total bilirubin (mg/dL)	0.80 ± 0.36	0.80 ± 0.36	0.83 ± 0.36	0.72 ± 0.33	< 0.001
ALT (U/L) (n = 113,969)	18.0 (13.0–27.0)	15 (11–21)	21 (15–29)	28 (18–43)	< 0.001
Serum creatinine (mg/dL)	0.81 ± 0.17	0.77 ± 0.17	0.86 ± 0.17	0.84 ± 0.18	< 0.001
Total cholesterol (mg/dL)	195.4 ± 33.8	187.8 ± 28.9	202.7 ± 36.3 ^a^	203.1 ± 36.7 ^a^	< 0.001
Triglycerides (mg/dL)	95 (67–141)	74 (57–96)	128 (87–178)	153 (108–222)	< 0.001
HDL cholesterol (mg/dL)	60.8 ± 16.6	67.9 ± 15.2	55.3 ± 15.4	50.6 ± 13.4	< 0.001
LDL cholesterol (mg/dL)	126.6 ± 32.6	117.3 ± 28.3	135.9 ± 33.9 ^a^	135.4 ± 33.9 ^a^	< 0.001
Fasting glucose (mg/dl)	94.9 ± 13.8	89.4 ± 5.9	97.7 ± 12.0	106.1 ± 23.5	< 0.001
HbA1c	5.48 ± 0.48	5.35 ± 0.25	5.53 ± 0.46	5.77 ± 0.82	< 0.001
Insulin	6.04 (4.24–8.56)	4.96 (3.59–6.64)	6.18 (4.64–7.84)	12.76 (11.20-15.51)	< 0.001
HOMA-IR	1.40 (0.95–2.05)	1.10 (0.78–1.49)	1.50 (1.10–1.92)	3.19 (2.77–3.99)	< 0.001
hsCRP (mg/L) (n = 88,275)	0.04 (0.03–0.08)	0.03 (0.02–0.06)	0.05 (0.03–0.09)	0.08 (0.04–0.15)	< 0.001
Systolic BP (mmHg)	109.5 ± 12.5	104.1 ± 9.4	114.0 ± 12.8	116.8 ± 12.7	< 0.001
Diastolic BP (mmHg)	71.0 ± 9.8	66.8 ± 7.2	74.8 ± 10.2	75.9 ± 10.1	< 0.001
Measured FEV1 (liter)	3.32 ± 0.68	3.42 ± 0.66	3.39 ± 0.65	3.22 ± 0.68	< 0.001
FEV1%	97.7 ± 10.7	98.6 ± 10.7	97.1 ± 10.7	96.0 ± 10.7	< 0.001
Measured FVC (liter)	4.04 ± 0.86	4.22 ± 0.83	4.16 ± 0.83	3.87 ± 0.86	< 0.001
FVC%	97.9 ± 10.7	98.7 ± 10.7	97.3 ± 10.5	96.4 ± 11.0	< 0.001
FEV1(L)/FVC(L) ratio	0.83 ± 0.06	0.84 ± 0.06	0.81 ± 0.06	0.82 ± 0.05	< 0.001
Diabetes	2,227 (2.0)	0 (0)	1,240 (3.1)	987 (5.6)	< 0.001
Hypertension	8,241 (7.2)	0 (0)	5,567 (13.8)	2,674 (15.2)	< 0.001

Data are presented as mean ± standard deviation, median (interquartile range), or number of subjects with percentage in parenthesis. We recorded numbers of subjects with available clinical parameters. Unless otherwise indicated, the available subject number was 114,143

^*^P values for one-way ANOVA test among three groups

^a^ No differences between the groups with the same footnotes in post-hoc analyses. Otherwise, all pairs of groups showed significant differences in post-hoc analyses

ALT = alanine aminotransferase; BMI = body mass index; BP = blood pressure; FEV1% = percent predicted forced expiratory volume in 1s; FVC% = percent predicted forced vital capacity; HDL = high-density lipoprotein; HbA1c = hemoglobin A1c; HOMA-IR = homeostasis model assessment of insulin resistance; hs-CRP = high-sensitivity C-reactive protein; IR = insulin resistance; LDL = low-density lipoprotein; MH = metabolically healthy; MS = metabolic syndrome

### Lung function among the three groups divided by metabolic health and IR status

Table 2 displays the comparison of lung function parameters between study groups after adjusting for age, sex, center, BMI, smoking (pack-years), alcohol consumption (g/day), moderate physical activity frequency (times/week), systolic BP, glucose, HbA1c, insulin, lipid profiles, liver enzymes, creatinine, and hsCRP. The MS with IR group had the lowest values of FEV1 (L), FEV1%, FVC (L), and FVC% but the highest FEV1/FVC ratio among the three groups (*P* < 0.001). All spirometric values were significantly different among groups (*P* < 0.001) except FEV1%, FVC%, and FEV1/FVC ratio between the MH and MS without IR groups.

**Table 2:** Adjusted mean values of lung function parameters in the study groups stratified by metabolic health and insulin resistance

	Category			*p* value by ANCOVA	Adjusted *p* value^a^		
	MH (n = 56,3111) (49.3%)	MS without IR (n = 40,255) (35.3%)	MS with IR (n = 17,577) (15.4%)		MH vs. MS without IR	MH vs. MS with IR	MS without IR vs. MS with IR
FEV1 (L)	3.372 ± 0.002	3.351 ± 0.003	3.288 ± 0.004	< 0.001	< 0.001	< 0.001	< 0.001
FEV1%	98.242 ± 0.059	98.178 ± 0.065	96.950 ± 0.107	< 0.001	1.000	< 0.001	< 0.001
FVC (L)	4.104 ± 0.003	4.084 ± 0.003	3.978 ± 0.005	< 0.001	< 0.001	< 0.001	< 0.001
FVC%	98.571 ± 0.058	98.645 ± 0.063	96.832 ± 0.105	< 0.001	1.000	< 0.001	< 0.001
FEV1(L)/FVC (L) ratio	0.8253 ± 0.0003	0.8247 ± 0.0003	0.8307 ± 0.0005	< 0.001	0.711	< 0.001	< 0.001

Data are presented as adjusted mean ± standard error. The multivariable model was adjusted for age, sex, center, and continuous variables with p *<* 0.05 in univariate analyses, comprising BMI, smoking (pack-years), alcohol consumption (g/day), moderate physical activity frequency (times/week), systolic blood pressure, glucose, HbA1c, insulin, lipid profiles, liver enzymes, creatinine, and high-sensitivity C-reactive protein

^a^Adjusted p value using the Bonferroni correction

FEV1% = percent predicted forced expiratory volume in 1 s; FVC% = percent predicted forced vital capacity; IR = insulin resistance; MH = metabolically healthy; MS = metabolic syndrome

### Comparison of clinical and laboratory parameters among groups stratified by metabolic health and IR status

Table 3 shows the comparison of clinical and laboratory characteristics among the three groups. Subjects in the MH group were younger and less likely to smoke and drink alcohol and more subjects were highly educated. In contrast, the MS with IR group showed significantly worse BMI, hepatic enzymes, lipid profiles, FBS, HbA1c, hs-CRP, insulin, and BP than the other two groups. Moreover, the proportions of subjects with FEV1% <80% and FVC% <80% were significantly higher in the MS with IR group compared to the other groups.

**Table 3:** Comparisons of demographic and clinical parameters according to metabolic health and insulin resistance status

	All Subjects (n = 114,143)	MH (n = 56,311) (49.3%)	MS without IR (n = 40,255) (35.3%)	MS with IR (n = 17,577) (15.4%)	*p* value
Age (≥ 40 years)	49,240 (43.1)	19,212 (34.1)	22,398 (55.6)	7,630 (43.4)	< 0.001
Sex (male)	65,342 (57.2)	23,430 (41.6)	29,751 (73.9)	12,161 (69.2)	< 0.001
Center (Seoul)	56,201 (49.2)	29,434 (52.3)	20,179 (50.1)	6,588 (37.5)	< 0.001
Obesity: BMI (≥ 25 kg/m^2^)	34,877 (30.6)	8,184 (14.5)	14,721 (36.6)	11,972 (68.1)	< 0.001
Current smokers	18,991 (16.6)	6,166 (10.9)	9,096 (22.6)	3,729 (21.2)	< 0.001
Heavy alcohol intake (> 20 g/day)	22,746 (19.9)	7,716 (13.7)	10,796 (26.8)	4,237 (24.1)	< 0.001
Regular exercise (≥ 3 times/week) (n = 113,742)	15,346 (13.5)	7,743 (13.8)	5,863 (14.6)	1,740 (9.9)	< 0.001
High education (≥ college education) (n = 111,747)	94,181 (84.3)	47,630 (86.2)	32,687 (83.1)	13,864 (80.7)	< 0.001
Elevated bilirubin (> 1.9 mg/dL)	1,564 (1.4)	799 (1.4)	622 (1.5)	143 (0.8)	< 0.001
Elevated ALT (> 40 U/L) (n = 113,969)	11,270 (9.9)	1,982 (3.5)	4,358 (10.8)	4,930 (28.1)	< 0.001
Elevated serum creatinine (> 1.2 mg/dL)	454 (0.4)	91 (0.2)	253 (0.6)	110 (0.6)	< 0.001
Hypercholesterolemia (≥ 220 mg/dL)	23,491 (20.6)	6,836 (12.1)	11,584 (28.8)	5,071 (28.9)	< 0.001
Hypertriglyceridemia (≥ 150 mg/dL)	25,290 (22.2)	0 (0.0)	16,177 (40.2)	9,113 (51.8)	< 0.001
Low HDL cholesterol^a^	12,759 (11.2)	0 (0.0)	8,093 (20.1)	4,666 (26.5)	< 0.001
High LDL cholesterol (≥ 159 mg/dL)	17,001 (14.9)	4,099 (7.3)	9,003 (22.4)	3,899 (22.2)	< 0.001
Hyperglycemia at fasting (≥ 100 mg/dl)	22,287 (19.5)	0 (0.0)	13,524 (33.6)	8,763 (49.9)	< 0.001
High HbA1c > = 6.5	2,144 (1.9)	2 (0.0)	827 (2.1)	1,315 (7.5)	< 0.001
Elevated hsCRP (> 0.5 mg/l) (n = 88,275)	2,246 (2.5)	795 (1.8)	811 (2.6)	640 (4.6)	< 0.001
High HOMA-IR ≥ 2.5	17,404 (15.2)	0 (0.0)	0 (0.0)	17,404 (99.0)	< 0.001
High insulin ≥ 25	623 (0.5)	0 (0.0)	0 (0.0)	623 (3.5)	< 0.001
High SBP ≥ 130	7,438 (6.5)	0 (0.0)	4,813 (12.0)	2,625 (14.9)	< 0.001
FVC% <80%	4,247 (3.7)	1,681 (3.0)	1,621 (4.0)	945 (5.4)	< 0.001
FEV1% <80%	4,878 (4.3)	1,925 (3.4)	1,924 (4.8)	1,029 (5.9)	< 0.001
FEV1(L)/FVC(L) ratio < 0.7	2,121 (1.9)	774 (1.4)	1,030 (2.6)	317 (1.8)	< 0.001

Data are presented as mean ± standard deviation, median and (interquartile range), or number of subjects with percentage in parentheses. We recorded subject numbers with available clinical parameters. Unless otherwise indicated, the available subject number was 114,143

^a^Low HDL was defined as *<* 40 mg/dL in males and *<* 50 mg/d/L in females

ALT = alanine aminotransferase; BMI = body mass index; BP = blood pressure; FEV1% = percent predicted forced expiratory volume in 1 s; FVC% = percent predicted forced vital capacity; HDL = high-density lipoprotein; HbA1c = hemoglobin A1c; HOMA-IR = homeostasis model assessment of insulin resistance; hs-CRP = high-sensitivity C-reactive protein; IR = insulin resistance; LDL = low-density lipoprotein; MH = metabolically healthy; MS = metabolic syndrome

### Odds ratios for impaired lung function according to metabolic health and IR status

Multiple logistic regression analysis was performed to determine the effects of MS and IR on lung function impairment (Table 4). After adjusting for only demographic variables of age, sex, center, smoking status, alcohol intake, regular exercise, and education level (model 1), ORs for FEV1% and FVC% (aOR = 1.362 (1.277–1.453) and 1.384 (1.291–1.483), respectively) were significantly higher in MS subjects compared to the MH (reference) group. However, MS was no longer a significant risk for lung dysfunction after adjusting for additional metabolic components of BMI, TC, LDL-C and HbA1c (model 2).

**Table 4:** Multiple logistic regression analysis of impaired lung function according to metabolic health and insulin resistance

	Model 1			Model 2			Model 3		
	OR (95% CI)	*p* value	*p* for trend	OR (95% CI)	*p* value	*p* for trend	OR (95% CI)	*p* value	*p* for trend
FEV1%<80%									
MH (reference)	1			1			1		
MS	1.362 (1.277–1.453)	< 0.001		1.108 (0.999–1.229)	0.053		1.103 (0.993–1.224)	0.067	
FVC%<80%									
MH (reference)	1			1			1		
MS	1.384 (1.291–1.483)	< 0.001		1.004 (0.895–1.127)	0.946		1.011 (0.901–1.136)	0.849	
FEV1(L)/FVC(L) ratio < 0.7									
MH (reference)	1			1			1		
MS	1.064 (0.967–1.172)	0.205		1.092 (0.939–1.26)	0.252		1.124 (0.966–1.307)	0.131	
FEV1%<80%			< 0.001			< 0.001			< 0.001
MH (reference)	1			1			1		
MS without IR	1.216 (1.132–1.305)	< 0.001		1.084 (0.996–1.180)	0.061		1.078 (0.975–1.192)	0.142	
MS with IR	1.667 (1.524–1.824)	< 0.001		1.429(1.279–1.597)	< 0.001		1.374 (1.205–1.566))	< 0.001	
FVC%<80%			< 0.001			< 0.001			< 0.001
MH (reference)	1			1			1		
MS without IR	1.191 (1.103–1.286)	< 0.001		1.080 (0.987–1.183)	0.095		1.000 (0.896–1.116)	0.998	
MS with IR	1.867 (1.698–2.053)	< 0.001		1.623 (1.443–1.826)	< 0.001		1.428 (1.237–1.647)	< 0.001	
FEV1(L)/FVC(L) ratio < 0.7			0.500			0.407			0.469
MH (reference)	1			1			1		
MS without IR	1.176 (1.061–1.303)	0.002		1.117 (0.986–1.265)	0.082		1.135 (0.982–1.312)	0.086	
MS with IR	0.977 (0.844–1.132)	0.757		0.902 (0.755–1.079)	0.261		0.896 (0.728–1.103)	0.300	

Model 1 was adjusted for age, sex, center, smoking status, alcohol intake, regular exercise, and education level. Model 2 was adjusted as in model 1 plus for metabolic components of BMI, total cholesterol, LDL-C and HbA1c. Model 3 was adjusted as in model 2 plus for variables with *p* < 0.05 in univariate analyses

CI = confidence interval; FEV1%=percent predicted forced expiratory volume in 1 s; FVC%=percent predicted forced vital capacity; IR = insulin resistance; MH = metabolically healthy; MS = metabolic syndrome; OR = odds ratio

To investigate the effects of these observed associations were mediated by IR in the same way, we estimated aORs for lung dysfunction in the three groups stratified by metabolic health and IR status. According to the fully adjusted logistic regression analysis (model 3), MS with IR was associated with decreased FEV1% and FVC% (aOR = 1.374 (1.205–1.566) and 1.428 (1.237–1.647), respectively), whereas MS without IR was not significantly associated with lung dysfunction. In contrast, the difference in aORs for OLF between groups was consistently not significant.

## Discussion

In the current cohort study, the label of MS, defined as presence of any MS component including IR, was not associated with lung dysfunction. In contrast, MS with IR was associated with decreased lung function, but this was not evident for MS without IR. This indicates that IR is a more important determinant for lung dysfunction than is MS. To the best of our knowledge, this study is the first to describe MS-related lung effects using a definition including IR as a criterion and supports the critical effect of IR on lung function.

Recent meta-analysis showed the harmful effects of MS on lung function [6]. However, we demonstrated that the association between MS and lung dysfunction is largely attenuated after adjustment for other metabolic parameters and inflammation markers that are not included in the criteria for MS but are related to lung health [38]. In contrast to previous studies, we used a less-strict definition in an apparently lung disease-free and middle-aged population, which resulted in shifting our subjects with MS to those with a low burden of metabolic abnormalities. These changes seemed to attenuate the effect of MS on lung function and may have contributed to the lack of statistical significance. Our differing results from previous studies may complicate conclusions on whether MS has a negative effect on lung function. However, it may provide a clear test of the association between MS and lung function that was missed in previous studies, because lung dysfunction is related to each metabolic parameter [9–26] and their intensity [9–15]. Therefore, the label of MS may not consistently be optimized to predict lung dysfunction but can be an artifact of the choice of factors used to define altered metabolism related to lung dysfunction.

In contrast, our findings support the role of IR in lung dysfunction and MS. IR is a multifaceted syndrome related to individual MS components [39]. In addition, lung dysfunction has been reported in individuals with IR [22, 23, 25], and impaired lung function predicts the development of IR [40]. Consequently, IR is thought to serve as a “primary link” between lung function impairment and MS [5, 22, 24, 28]. However, concurrent definitions of MS appear to be only modestly successful in identifying IR. Moreover, the greater is the number of MS components present, the higher is the prevalence of IR [29]. The disagreement between MS definition and presence of IR could lead to limitations of concurrent definitions of MS for explanation of MS-related lung dysfunction, especially when using a less strict definition of MS, as in the current study. This could explain our lack of significance in the relationship between MS and lung function and MS-related lung effects according to presence of IR.

Although the precise mechanisms by which IR affects lung function remain unclear, changes in IR-related factors such as free fatty acids (FFA), inflammatory cytokines, mitochondrial dysfunction, and adipokines have been suggested [5, 39, 41]. The increase in FFA reduces glucose utilization and induces abnormal fat metabolism in skeletal muscle. IR also leads to smaller and fewer mitochondria, more of which demonstrate impaired function, which may reduce mitochondrial ATP production and skeletal muscle strength [41]. As forced respiration during spirometry requires respiratory skeletal muscle contraction, IR could mediate a decrease in lung function. Additionally, elevated FFA and hypo-adiponectinemia increase inflammatory cytokines. This could contribute to activation and adhesion of inflammatory cells to the pulmonary capillary endothelium, leading to damage to the airways and a decrease in lung function [5]. Last, hyperinsulinemia could exert a direct negative effect on the airway through airway epithelial damage and airway smooth muscle proliferation [5]. Taken together, these findings indicate IR as an explanatory mechanism leading to lung dysfunction.

Interestingly, we found no association between OLF and MS, regardless of the presence of IR. The reasons for this result are not fully understood. The effect of MS on OLF is controversial, with one study finding a negative correlation [12] and others finding the opposite [18, 20], although most found no association [9–11, 14, 16, 22, 24, 28], as in our study. Also, OLF is associated with systemic inflammation [42] but not IR [28]. It seems that the major effect of metabolic derangement is on the lung tissue, with slight effect on airway diameter. Previous studies have also shown an association of OLF with systemic inflammation [42] but not MS [24, 28]. Metabolic derangement could not be associated with OLF, especially in our cohort with median CRP close to the upper normal limit to define systemic inflammation. Moreover, functional debility of the airways might have gone undetected on screening spirometry in our healthy subjects because OLF predominantly reflects obstruction of large airways. These seem to attenuate the relationship between MS and OLF, and careful consideration is required when assessing OLF based on screening spirometry, especially in healthy young and middle-aged subjects.

The current study demonstrates a clear association of modifiable IR with lung dysfunction. Lung dysfunction is associated with respiratory and non-respiratory diseases as well as their risk of mortality [1–3]. Therefore, this study has an important strength in that early detection and intervention for IR can reduce mortality risk related to respiratory and other non-respiratory complications. Other strengths of our study are a large sample size, standardized spirometric techniques, and extensive data on potential confounders that increased precision and permitted sufficient statistical power.

However, our study has several limitations. First, a cross-sectional study design tends to incur uncertainty regarding the temporal sequence of exposure–outcome relations. Thus, further longitudinal follow-up studies are needed to validate our findings. Second, our results were obtained from middle-aged asymptomatic and relatively healthy Korean adults. Therefore, our findings cannot be generalized to other demographic populations. In addition, there might be considerable differences in the outcomes based on MS definition [27]. Consequently, the current results should be interpreted cautiously in accordance with racial differences and the criteria used to define MS. Finally, it is possible that some subjects had undetected cardio-metabolic and pulmonary disease because of the questionnaire-based collection of medical histories. This might have altered outcomes, as these subclinical diseases can contribute to lung dysfunction especially among individuals with MS or IR.

In conclusion, the effect of MS on lung function could be altered according to the presence of IR. Therefore, IR is a more important determinant for lung dysfunction than is the label of MS. Our study supports and extends previous findings that IR could be a critical component in mediating the association between MS and lung dysfunction. However, longitudinal follow-up studies and prospective interventional studies are needed to validate our findings.

## Data Availability

The data are not publicly available because of institutional review board restrictions (the data were not collected in a way that could be distributed widely). However, the analytical methods are available from the corresponding author upon request.

## List of abbreviations

ANCOVA: analysis of covariance
aOR: adjusted odds ratio
BMI: body mass index
BP: blood pressure
CI: confidence interval
FBS: fasting glucose level
FEV1%: percent predicted forced expiratory volume in 1 s
FFA: free fatty acids
FVC%: percent predicted forced vital capacity
HbA1c: glycated hemoglobin
HDL-C: high-density lipoprotein cholesterol
HOMA-IR: homeostasis model assessment of insulin resistance
hsCRP: high-sensitivity C-reactive protein
IR: insulin resistance
IQR: interquartile range
LDL-C: low-density lipoprotein cholesterol
MH: metabolically healthy
MS: metabolic syndrome
OLF: obstructive lung function
SD: standard deviations
TC: total cholesterol
TG: triglycerides
